# Sexual Differences in Cell Proliferation in the Ventricular Zone, Cell Migration and Differentiation in the HVC of Juvenile Bengalese Finch

**DOI:** 10.1371/journal.pone.0097403

**Published:** 2014-05-19

**Authors:** Qiong Chen, Xuebo Zhang, Yueliu Zhao, Xin Zhou, Lina Sun, Shaoju Zeng, Mingxue Zuo, Xinwen Zhang

**Affiliations:** 1 Beijing Key Laboratory of Gene Resource and Molecular Development, Beijing Normal University, Beijing, China; 2 College of Life Sciences, Hainan Normal University, Haikou, China; 3 Department of Laboratory Medicine, The Third Affiliated Hospital, Guangzhou Medical University, Guangzhou, China; Pennsylvania State University, United States of America

## Abstract

Song control nuclei have distinct sexual differences and thus are an ideal model to address how brain areas are sexually differentiated. Through a combination of histological analysis and electrical lesions, we first identified the ventricle site for HVC progenitor cells. We then found that there were significant sex differences in the cellular proliferation activity in the ventricular zone of the HVC, the number of migrating cells along the radial cells (positive immunoreactions to vimentin) and differentiation towards neurons. Through co-culturing of male and female slices containing the developing HVC in the same well, we found that the male slices could produce diffusible substances to masculinize the female HVC. By adding estrogen, an estrogen antagonist, brain-derived neurotrophic factor (BDNF) or its antibody into the culture medium, separately or in combination, we found that these diffusible substances may include estrogen and BDNF. Finally, we found that 1) estrogen-induced BDNF upregulation could be detected 48 hr after estrogen treatment and could not be blocked by a vascular endothelial growth factor (VEGF) receptor inhibitor and 2) the amount of VEGF mRNA expressed in the developing HVC and its adjacent area did not display any significant sex differences, as did the distribution of VEGF and laminin-expressing endothelial cells in the developing HVC. Because these findings are largely different from previous reports on the adult female HVC, it is suggested that our estrogen-induced BDNF up-regulation and the resultant sexual differentiation might not be mediated by VEGF and endothelial cells, but instead, may result from the direct effects of estrogen on BDNF.

## Introduction

Sexually dimorphic brain areas have been documented in a variety of vertebrates, of which song control nuclei in oscine species are the most obvious [1]. Song control nuclei thus provide an ideal model to address how brain areas are sexually differentiated and explore the mechanism underlying the process [2].

In mammals, tremendous progress has been made in elucidating sexual differentiation. It has been demonstrated that the testis-determining gene, the sex-determining region Y on the Y chromosome, causes the embryonic undifferentiated gonad to develop into a testis rather than an ovary, and the testes, in turn, secrete steroid hormones, which direct specific cells throughout the body, including some brain areas, to develop in a male-typical fashion [3], [4], [5].

Although dosage compensation occurs in insects, mammals and many other vertebrates, generally, it does not occur in birds with homogametic males (ZZ) and heterogametic females (ZW) [6]. To date, some reports have indicated that the sexual differentiation of the brain areas in songbirds differs largely from that reported in mammals [7], including incomplete sex-reverse of masculinization in female birds with estradiol (E2) treatment [8] and unsuccessful prevention of masculine development in male birds by blocking testicular hormones [9]. Because one to three weeks are needed for proliferated cells in the ventricular zone to migrate into the song control nuclei [10], [11], [12], sexually dimorphic proliferation and migration en route to their final destination or differentiation in their targets might affect sex differences following the net addition of neurons [13], [14], [15], [16], [17]. To our knowledge, it has not been established how sexual differences during the development of a song control nucleus, high vocal center (HVC), are affected by the aforementioned activities in juvenile birds, and the underlying mechanisms have not been established.

To address these issues, we first identified the ventricle site for HVC progenitor cells, whose accurate position still remains unclear, although some reports have concerned it [10], [13], [18]. We then studied whether there were sexual differences in the cell proliferation in the HVC ventricle site at posthatching 15 days (P15) when HVC progenitor cells are generated in large scale [14], [15], [19], and in the number of cells migrating towards HVC along radial glia fibers, or differentiating toward neurons within HVC. To exclude the effect of gonad-derived steroids *in vivo*, the corresponding studies were also performed *in vitro*. Two lines of results both revealed that proliferation in the ventricle site, and migration or differentiation after generation all differed largely between the two sexes. We further found that co-culturing of male and female brain slices could result in a significant “masculinizing” of female HVC. Although both estrogen and BDNF have been demonstrated to be able to increase the number of newborn HVC cells [20], [21], the mechanism has not yet been known. By performing serial experiments, we found that both estradiol and BDNF had effect on the cell proliferation in the ventricular zone, and on the cell differentiation in the developing HVC, and that the increase of BDNF in HVC was not VEGF and vascular endothelial cells-mediated as reported in adult HVC of female canaries [21], [22], but was estrogen dependent.

## Materials and Methods

### Songbirds and the Determination of the Location of the Developing HVC at 15 Post-hatch Days

Bengalese finches (*Lonchura striata*), which belong to the same family (Estrildidae) as the zebra finch *(Poephila guttata)* [23], were bred and raised in a breeding colony or bought from local markets, with 4–6 birds living in a cage (50 cm×62 cm×38 cm). According to some previous reports [24]–[27], the sexual differentiation of song nuclei and their adult sizes, the establishment of neural connectivity among song nuclei, and electrophysiological responses such as in HVC are proximately similar to those in the zebra finch. The birds were kept under a 14/10 h light/dark cycle at 19–25°C. Seeds and fresh water were available ad libitum, supplemented with a mixture of cooked eggs. All experiments were performed in accordance with the guidelines for animal care issued by the Beijing Animal Administration Committee. The protocol was reviewed and approved by the Animal Management Committee of College of Life Sciences, Beijing Normal University.

For neural tract tracing, male and female Bengalese finches at P15 were euthanized with an overdose of Equithesin and perfused transcardially with ice-cold PBS (phosphate-buffered saline, pH 7.4), followed by 4% paraformaldehyde in PB (phosphate buffer 0.1 M, pH 7.3). After the sex of the birds was determined by inspection of the gonads, the brains (n = 3 for each sex) were post-fixed in the same fixative for 24 h and then immersed in 30% sucrose overnight at 4°C. The hemispheres were cut with a vibratome into 600-µm sagittal slices. Under a stereoscopic microscope (Scoptic, China), one to six crystals of DiI were stabbed into Area X by using a tungsten needle. Area X is located under the dorsal top of the lamina pallio-subpallialis (LPS) which could be clearly identified under a stereoscopic microscope. The slices were then immersed in 4% paraformaldehyde, and the dye was allowed to diffuse for 50 days at 35°C. Then, the slices were cut into 10-µm sections with a cryostat. Because the axons from Area X-projecting neurons have already innervated Area X at P15, Area X-projecting neurons will be retrogradely labeled after DiI is delivered to Area X, leading to the developing HVC being distinguishable [28], [29].

### Electrical Lesions of the “hot spot” of Cell Proliferation at the End of Dorsal Ventricular Zone

For the electrical lesion, the birds at P15 were deeply anesthetized by pentobarbital and held in a stereotaxic head holder. The stereotaxic point 0.0 was defined both for the anteroposterior and the mediolateral axes as the branch point of the sagittal sinus that lies just anterior to the rostral tip of the cerebellum. The “hot spot” for cell proliferation at the end of dorsal ventricular zone (which is relatively close to the HVC, and is thus most probably a potential site for HVC progenitor cells [13], [19]) was electrically injured at either side of the hemispheres with the coordinate (Lateral: 1.0 mm, Anterior: 3.2 mm, Deep: 1.3 mm) for 90 s at 100 µA, whereas the other side was kept intact, and used as control. After producing the lesion, the animals were returned to the colony in which they were kept with their family until P60, when the HVC was well developed [30]. Then, the birds were sacrificed, and the sizes of HVC were compared between the lesioned and intact hemispheres (n = 4 males).

### Immunohistochemistry and [^3^H]-thymidine Autoradiography

Bengalese finches at P15 or at adult (>90 days) were killed and perfused as described above. After the brains were embedded in Jung compound (Leica), they were sectioned on a freezing microtome (Leica) into 10-µm sections. Every fifth sagittal section was mounted on a gelatin-coated slide. The sections were pretreated with 0.3% H_2_O_2_ in 80% methanol PBS and then with 3% horse serum in PBS with Triton X-100 (0.05%). The sections were then incubated at 4°C overnight with the following primary antibodies, respectively: rabbit anti-VEGF (1∶200, Neomarker, RB-222-P), rabbit anti-laminin (1∶200, Sigma, L9393), anti-HuC/HuD neuronal protein (1∶800, Molecular Probes, 16A11), mouse anti-VM (1∶200; Chemicon, VM 3B4), anti-NeuN (1∶500, Chemicon, MAB 377), anti-GFAP (1∶800; Chemicon, AB16901), sheep anti-BDNF (1∶200, Chemicon, AB1513P) and rabbit anti-TrkB (Santa Cruz Biotechnology, sc-12).

The specificity of the above used antibodies was verified in previous reports on songbirds or chickens, including anti-VEGF, anti-laminin and anti-HuC/HuD [22], anti-NeuN [18], anti-BDNF [20], anti-TrkB [31] and anti-VM or anti-GFAP [32]. The antibody specificity was also confirmed from our staining patterns which were largely similar to related reports. The sections were then incubated for 2 hr at room temperature with the secondary antibody: biotinylated anti-mouse IgG (1∶500, Vector), rabbit IgG or sheep IgG (corresponding to the primary antibody) and avidin-biotinylated peroxidase complex (1∶150; Vector Elite Kit). Diaminobenzidine (DAB; Sigma; 0.05%) was used as a chromagen to visualize the distribution of the antibody.

Each of Bengalese finches at P15 received an intramuscular injection of [^3^H] thymidine (2.5 mCi/g dose; specific activity, 6.7 Ci/mmol; New England Nuclear) twice a day for two consecutive days. The birds were allowed to survive for different times: 2 h (labeled cells do not begin to migrate out of ventricular zone, [19]), 5 d (labeled cells begin to migrate toward their targeting area, [11]) and 10 d or 25 d (labeled cells have reached their targeting area, [10], [12], [17]). As described above, 10-µm sections were cut, and the sections were then covered with a layer of emulsion. After the sections were exposed for four weeks in lightproof boxes at 4°C (Kodak, NTB-2 emulsion), they were then developed in D19 and counterstained with cresyl violet. Only cells with a clear neuronal morphology (darkly stained nucleoli or relatively large cytoplasm) were regarded as neurons. A neuron was considered labeled with [^3^H]-thymidine if autoradiographic grains overlying its nucleus were over 10× background density (Background grain counts were determined for each specimen) [19]. For double labeling, the sections were first processed for immunohistochemistry, and then [^3^H]-thymidine autoradiography, as described above. Identification of relevant brain regions was based on zebra finch atlas [33].

### Brain Slice Preparation and Culture

**Figure 1:**
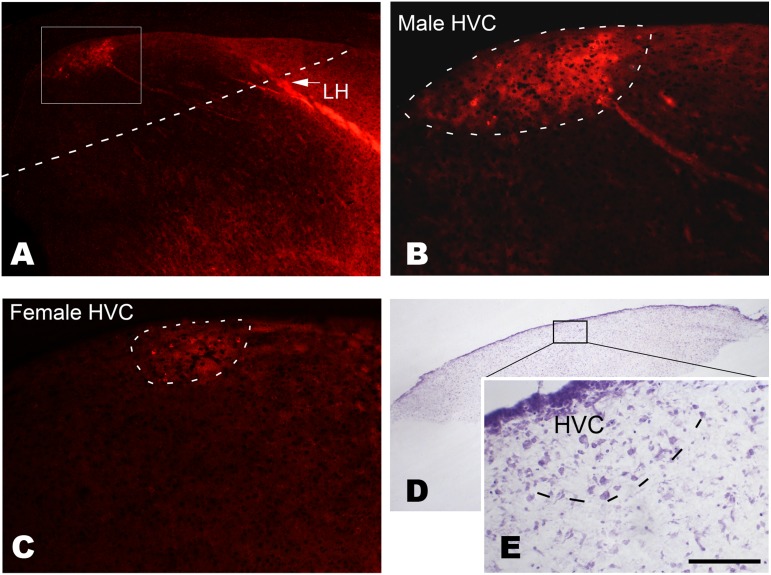
Determination of the location of the developing HVC at post-hatch day 15. Following the injection of DiI into Area X, retrograde labeled cells are observed in the developing HVC of males (A and B) or females (C). The cells are located caudally to the lamina hyperstriatica (LH). D and E: A Nissl-stained cultured brain slice containing the developing HVC, which has many relatively larger cells compared with its surrounding areas (E). The cultured brain slice is obtained by cutting along the dashed line shown in A. The boxed area is amplified in B. Scale bar = 350 µm (A), 100 µm (B and C), 500 µm (D) and 75 µm (E).

Bengalese finches were anesthetized on P15 and then perfused transcardially with sterile ice-cold Hanks’ balanced solution (HBSS) supplemented with 5.5 g/L D-glucose (40 U/ml penicillin and streptomycin, pH 7.2). The hemispheres were cut with a vibratome into 400 µm slices parasagittally, yielding 2–3 slices per hemisphere containing the presumptive HVC. The explant containing the putative HVC and adjacent parenchyma was obtained by cutting along the dashed line (shown in Figure 1A). The cut line was located at the caudal hyperpallium lamina (LH), which could be clearly distinguished in the brain slices. The explants were transferred into cold HBSS and incubated for 90 min at 4°C and then placed on 25-mm porous culture inserts with a 0.4-µm pore size (NUCN, 137060) with six explants per insert in a 6-well plate. They were then cultured at 37°C in an humidified atmosphere with 5% CO_2_ in 1.8 ml of nutrient medium (DMEM: F-12 HAM 1∶1, 100 µg/ml L-ascorbic acid, 2 mM L-glutamate, penicillin, streptomycin and 10% new born calf serum). The culture medium was generally changed every third day.

To label the proliferating cells, BrdU (an analogue of [^3^H]-thymidine) was added to the culture medium (10 µΜ), but it was only allowed to be incorporated into the proliferating cells for 24 h, and then washed out. In addition, to study the effect of estrogen, BDNF, or VEGF on the sexual differentiation of HVC, 17-β-estradiol (Sigma, 20 ng/ml), human recombinant BDNF (10 ng/ml, Sigma, B3795), anti-BDNF antibody (2 µg/ml, Chemicon, 1513P), tamoxifen (5 µΜ in ethanol, a competitive inhibitor of the estrogen receptor, Merck, 579002), or VEGFR2 kinase inhibitor 1 (10 µΜ in DMSO, Calbiochem, 676480) was added to DMEM-F12 based medium (DMEM: F-12 HAM 1∶1, 100 µg/ml L-ascorbic acid, 2 mM L-glutamate, penicillin, streptomycin, N2, B27). These agents have been reported to use in songbirds, and the above dosages were largely based on these reports [34: for 17-β-estradiol and tamoxifen; 22: for the VEGF receptor inhibitor, and 20: for human recombinant BDNF and anti-BDNF], as well as on our previous preliminary experiment. To exclude the serum effect, the DMEM-F12 based medium was serum-free. After culture (the time ranged from 24 h to 5 d), the explants were either collected to extract mRNA or proteins, or fixed with 4% paraformaldehyde overnight, and then cut into 10 µm sections to perform immunohistochemistry.

We also cultured a small male or female explant (donor) with a relatively large male or female brain slice (recipient) in the same well for 24 h, both of which contained the developing HVC and were from the birds at P15. To label proliferating cells, BrdU (10 µΜ) was added to the culture medium. The donor was about 0.5×1.0×0.4 mm (as small as possible, to be readily affected by the substances diffused from the recipient), and the recipient was about 2.0×3.0×0.4 mm (two principle targets of HVC i.e., RA and Area X were not included). These two explants were cultured together in physical contact, with the developing HVC oriented in the same dorsoventrical direction.

### Immunohistochemistry in the Cultured Explants

Immunohistochemistry in the cultured explants was mainly performed following the procedure described above, and the exceptions are detailed as follows. For BrdU/Hu immunofluorescent double-labeling, the sections were first incubated in rat anti-BrdU antibody (BD Biosciences, 347580) and then donkey anti-rat IgG (H+L) conjugated to Texas red (1∶100, Jackson Immunoresearch). The sections were incubated with a mouse anti-Hu C/D antibody (1∶800, Molecular Probes) and then goat anti-mouse IgG (H+L) conjugated to Alexa Fluor 488, with minimal cross-reaction to rat, human, bovine, horse and rabbit serum proteins (1∶100, Molecular Probes). The specificity of the primary antibodies was verified in previous reports: [21] for anti-BrdU, and [22] for anti-Hu. The sections were finally mounted in glycerin.

### Quantitative-RT PCR

Total RNA was isolated from the above cultured explants by using TRIZOL (Invetrogen) and was then reverse-transcribed using reverse transcriptase (Takara). The concentration of each cDNA was determined by measuring the absorbance at 260/280 nm. Real-time PCR was performed with an ABI 7500 Sequence Detection System (Applied Biosystems), using the SYBR Green Mix kit with 10–20 nM cDNA template (2–4 µl), and 0.2 µΜ each primer (20 µl total volume):

BDNF forward, 5′-AGAAGCCAGTCTAAGAGGAC-3′ and.

reverse, 5′-AAGTGTCTGCCAACGATGTC-3′;

TrkB forward, 5′-CCATGGTATCAGCTCTCAAACAAT-3′ and.

reverse, 5′-TCATACACTTCCTTTGGGCATGT-3′;

VEGF forward, 5′-TGCGTCGAAGACGTCCTGTTA-3′ and.

reverse, 5′-CATCTCATCAGAGGCACACA-3′; and.

CD31/PECAM-1 forward, 5′- TTGGGCAGTGTTCCGTAT-3′ and.

reverse, 5′- GCCAGGCTGCTAAGATGA-3′.

β-actin was used as a control:

forward, 5′-TTGGCAATGAGAGGTTCAGGT-3′ and.

reverse, 5′-TACGGATGTCCACATCACACT- 3′.

The cycling conditions were 50°C for 2 min, 95°C for 10 min, and 40 cycles at 95°C for 15 s and 60°C for 1 min. Each sample was evaluated four times. The primers were designed with reference to the reported sequences of zebra finch which could be found in NCBI database. Only a single band with the expected length (150–300 bp) was obtained for each of the above studied genes.

### Images, Cell Counting and Statistical Analyses

Images in bright-fields were captured with a digital camera (Spot Enhance 2e; Diagnostic Instrument, Corp., USA) attached to the BH-2 microscope (Olympus, Japan), whereas those for fluorescent analysis were captured with a digital camera attached to an IX-70 fluorescent microscope (Olympus). The boundaries of the adult male HVC were not difficult to be identified on the basis of cell size and packing density within HVC, which are largely different from their surrounding regions. Although juvenile HVC, and adult female HVC also showed the above features, they were less apparent. However, with the help of neural tract-tracing, the location and the sizes of HVC could be determined in either male or female Bengalese finch even at P15 (Fig. 1A–C). In addition, some anatomical features, including cell size or the packing density in HVC, and its relative position to other structures such as LH and the ventricle were also helpful to determine HVC location and sizes.

The density of the labeled cells (per mm^2^) was calculated as the ratio of the number of labeled cells to the size of the examined areas. During these analyses, the experimenter was blind to the sex and the experimental treatments. Statistical analyses were performed using the SPSS 11.5 software package. Student’s t-test was used to compare the differences between the two groups under the same experimental condition. One-way ANOVA was conducted to compare the differences among the groups undertaken several treatments, and two-way ANOVA was adopted to examine the effect of gender and different age or culture time on the studied measurements such as the number of [^3^H]-thymidine-labeled cells. Before use of ANOVA, the distributions of dependent variable were tested for normality, and homogeneity of variances was assessed for equality of error variances (Levene’s test). Statistical significance and extreme significance were set at P<0.05 and p<0.001, respectively.

## Results

### Determining the Location of the Developing HVC on Post-hatch Day 15

Following the delivery of DiI to Area X, labeled fibers were observed arising from Area X. They coursed dorsocaudally and entered the lamina hyperstriatica (LH; Fig. 1A). Although the developing male HVC was obviously larger than the female HVC, they were both located in a similar brain position (about 1 mm caudal to the LH), and some labeled cells were observed in both sexes (Fig. 1A–C).

### Identifying the Ventricle Site for HVC Progenitors

**Figure 2:**
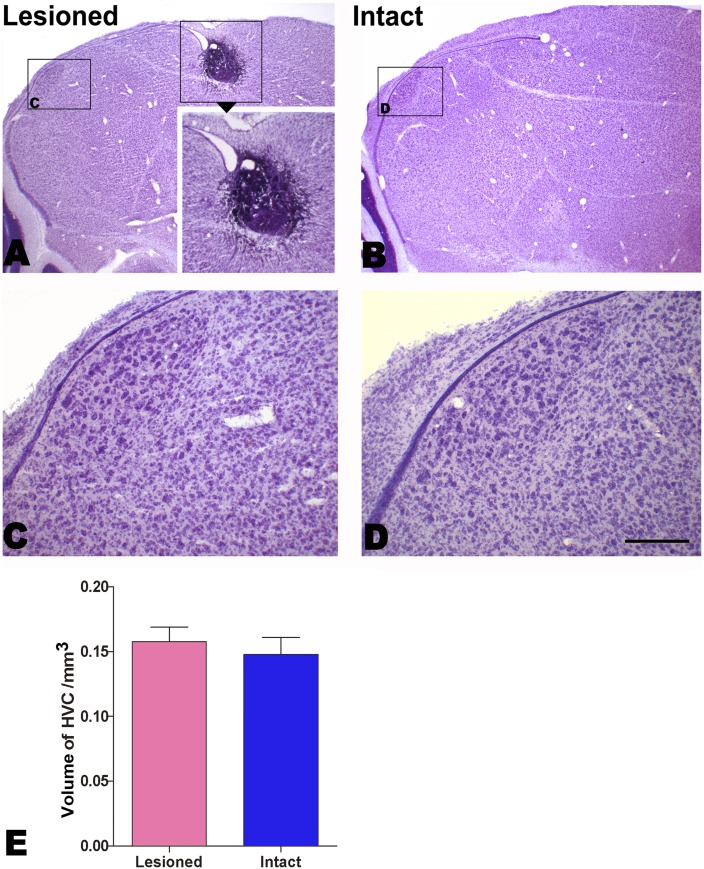
Determination of whether the dorsal proliferation “hot spot” is the ventricle site for HVC progenitors. A: The dorsal “hot spot” is electrically injured. B–E: No significant difference is detected in the HVC volume between the lesioned and intact hemispheres. The boxed areas in A and B are magnified in C and D, respectively. Scale bar = 1 mm (A, B), 500 µm (insert in A) and 200 µm (C, D).

As the diameters of the damaged sites were approximately 250 µm, which covered the “hot spot” itself [13], and its adjacent parenchyma, and no cells could be found in the damaged sites [Fig. 2A], the “hot spot” had to be damaged by the lesions. Following the lesions, there was no significant difference in the HVC volume between the injured and intact hemispheres (*n* = 4, t = –0.832, *P* = 0.418; Fig. 2A to E). Therefore, our results exclude the possibility of the hot spot as a potential site for the production of HVC progenitors. Thus, our study focused only on the site in the ventricular zone overlying the HVC [18].

### Sexual Differences in Cellular Proliferation in the Ventricle Site for HVC Progenitors, Migration and Differentiation in the HVC

**Figure 3:**
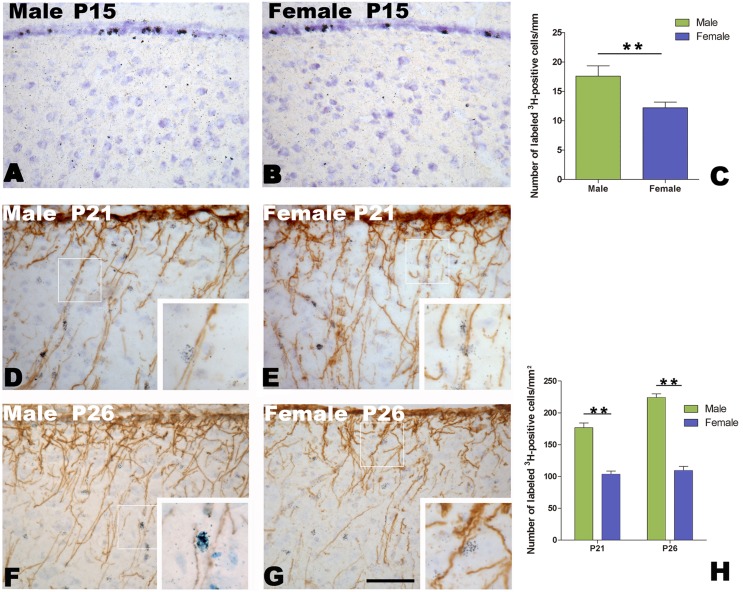
Proliferating cells in the ventricular zone and the migration of newborn cells along the radial glia fibers. A and B: [^3^H]-thymidine-labeled cells in the ventricular zone overlying the developing HVC after two hours administration of [^3^H]-thymidine at post-hatch day 15 in a male (A) and female (B). C: Comparison of the number of [^3^H]-thymidine-labeled cells per mm along the ventricular zone between the two sexes. D–H: The migration of [^3^H]-thymidine-labeled cells along vimentin-immunoreactive radial glia fibers within the developing HVC in males (D and F) and females (E and G) at post-hatch day 21 or 26. H: Comparison of the number of [^3^H]-thymidine-labeled cells along radial glia fibers per mm^2^ within the developing HVC between the two sexes. ** indicates *P*<0.001. Scale bar = 100 µm (A, B, D–G) and 40 µm (all inserts).

The proliferative epithelium examined here is what has classically been referred to as the ventricular zone, an epithelial cell layer adjacent to the lateral ventricle [35]. Our result showed a significant difference in the number of labeled cells per mm in the ventricular zone overlying the developing HVC at P15 (n = 6 for each sex, t  =   = –3.212, *P* = 0.02, male/female ratio = 1.42; Fig. 3A–C). Following the method used in a previous report [11], we compared the number of [^3^H]-thymidine-labeled cells along radial glia fibers (immunoreactive for vimentin) in the developing HVC at 6 d (P21) and 10 d (P26) after administration of [^3^H]-thymidine.

As shown in Fig. 3D–H, vimentin-immunoreactive radial glia fibers arose from the ventricular wall, with perpendicular orientation to the ventricular zone, and then penetrated into the parenchyma, including the developing HVC. These radial glia fibers disappeared once the HVC was well developed (cell migration is complete, data not shown). Two-way ANOVA showed that the number of [^3^H]-thymidine-labeled cells along radial glia fibers per mm^2^ in the HVC had significant differences between the two sexes (*F*_(1, 20)_  = 143.368, *P*<0.001. male/female ratio = 1.71 at P21, and 1.96 at P26), but not between the two age groups (*F*_(1, 20)_  = 7.317, *P* = 0.435) (Fig. 3F).

**Figure 4:**
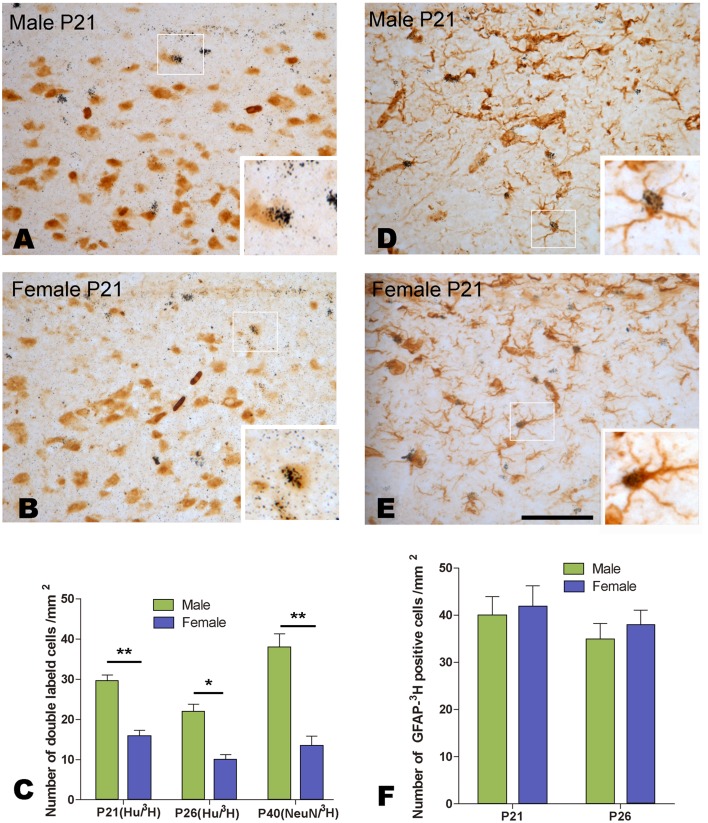
Cells double-labeled with [^3^H]-thymidine and Hu or GFAP within the developing HVC. A–E: Double-labeling with [^3^H]-thymidine and Hu (A and B) or GFAP (D and E) at post-hatch day 21. C: Comparison of the number of cells double-labeled with [^3^H]-thymidine and Hu (at post-hatch day 21 or 26) or NeuN (at post-hatch day 40) between the two sexes. F: The comparison of the number of cells double-labeled with [^3^H]-thymidine and GFAP (at post-hatch day 21 or 26). * indicates *P*<0.05, and ** indicates *P*<0.001. Scale bar = 100 µm (A, B, D, E) and 40 µm (all the inserts).

We also compared the density of the double-labeled cells for [^3^H]-thymidine and Hu in the developing HVC, and found that there were significant differences between the two sexes (*F*_(1, 20)_  = 22.455, *P*<0.001), and between the two different age groups (*F*_(1, 20)_  = 10.902, *P* = 0.001) (Fig. 4A–C). However, the density of the double-labeled cells for [^3^H]-thymidine and GFAP did not show any significant differences between the two sexes (*F*_(1, 20)_ = 0.273, *P* = 0.607), and the two different age groups (*F*_(1, 20)_ = 7.113, *P* = 0.445) (Fig. 4D–F). Our study further showed that the density of the cells double-labeled with [^3^H]-thymidine and NeuN (marker for mature neurons) in HVC at P40 (25 d after administration of [^3^H]-thymidine) was also significantly different between the two sexes (male/female ratio = 3.18, t = 7.881; *P*<0.0001; Fig. 4C).

### Sexual Differences in the Proliferative Activity in the Ventricle Site for HVC Progenitors, Migration and Differentiation in the Developing HVC In vitro

To exclude the effect of gonad-derived steroids *in vivo*, the corresponding studies above were also performed *in vitro*. The brain slices containing the developing HVC were cut as shown in Fig. 1 A (the cut line was just located in the obvious end of LH, which could be observed in the brain slices by the naked eye).

### Cellular Proliferation in the Developing HVC In vitro

**Figure 5:**
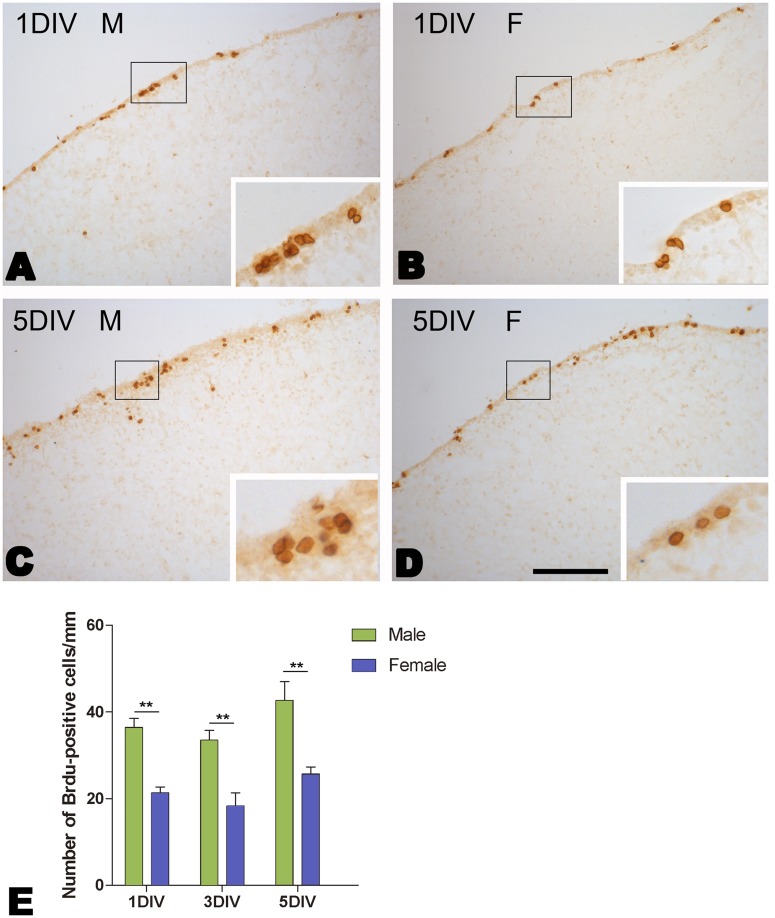
BrdU-labeled cells within the ventricular zone overlying the developing HVC in cultured brain slices at post-hatch day 15. A–D: BrdU-Labeling after the explants are cultured for one and five days *in vitro* (DIV) after BrdU addition. E: The comparison of the number of BrdU-labeled cells per mm along the ventricular zone after the explants are cultured for one, three and five days *in vitro* (DIV) after BrdU addition. ** indicates *P*<0.001. Scale bar = 400 µm (A–D) and 100 µm (all the inserts).

To assess the sexual differences in the proliferating activity in the ventricular zone overlying the developing HVC, we compared the rates of BrdU incorporation in the brain slices cultured for different time (1 d, 3 d and 5 d). For each group, BrdU was added to the culture medium on the last day before the harvest (only 24 h for BrdU incorporation). The brain slices in each group were averagely collected from 6 birds, and the number of the brain slices was around 30 (1 d: male, n = 30; female, n = 35; 3 d: male, n = 30; female, n = 36; 5 d: male, n = 36; female, n = 36). As shown in Figure 5C–D, most BrdU-labeled cells were located in the ventricular zone overlying the developing HVC, and more labeled cells appeared in the male brain slices. Two-way ANOVA analysis indicated significant differences in the number of BrdU-labeled cells per mm along the ventricular zone between the two sexes (*F*_(1, 198)_  = 46.265, *P*<0.001), but not among the groups with different culture time (*F*_(2, 198)_  = 2.489, *P* = 0.088) (Fig. 5E). These data indicated that the ventricular zone overlying the developing HVC still remained sexual differences in the proliferating activity after 5 d *in vitro*.

### Co-culture of Male and Female Explants Containing the Developing HVC

**Figure 6:**
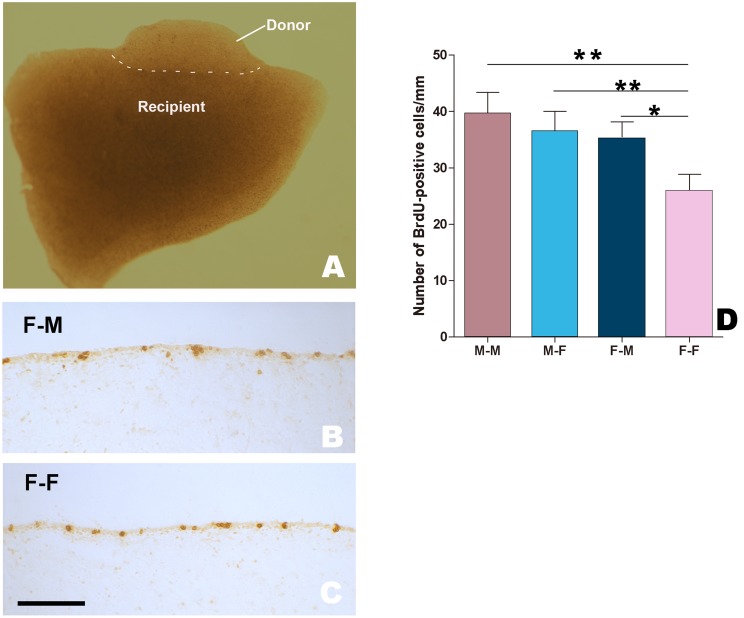
Two explants containing the developing *HVC* after 24 h co-culture *in vitro.* A: A small explant (donor) is close to a larger brain slice (recipient) with the same direction of the developing *HVC*. Both brain slices contain the developing HVC. B and C: BrdU-labeled cells within the ventricular zone overlying the developing HVC in a female tester co-cultured with a male (B) or female recipient. D: Comparison of the number of BrdU-labeled cells per mm along the ventricular zone in several cases of co-culture. * indicates *P*<0.05, and ** indicates *P*<0.001. Scale bar = 500 µm (A) and 100 µm (B, C).

We examined the number of BrdU-labeled cells in the ventricular zone overlying the developing HVC (Fig. 6B and C), and found that it was significantly different among the studied groups (F_(3, 49)_ = 76.89, p = 0.032). It was higher in the female donor when co-cultured with the male recipients (n = 14 from 5 birds) than that co-cultured with the female recipients (n = 12 from 4 birds; *P* = 0.003, Fig. 6B–D). However, the number of BrdU-labeled cells was not significantly different in the co-culture of a male donor with either a male (n = 12 from 4 birds) or female (n = 11 from 3 birds) recipient (Fig. 6D; P>0.05).

### Newborn Cell Migration and Differentiation in the Developing HVC In vitro

**Figure 7:**
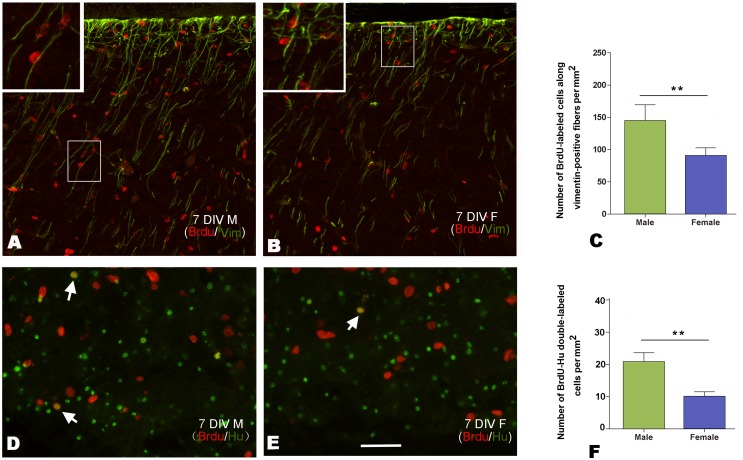
Newborn cell migration and differentiation in the developing HVC. A and B: Migration of BrdU-labeled cells along vimentin-immunoreactive radial glia fibers in the male (A) and female (B) after culture for seven days *in vitro* (7 DIV). C: Comparison of the number of BrdU-labeled cells along radial glia fibers per mm^2^ within the developing HVC between the two sexes. D and E: Cells double-labeled with BrdU and Hu in the developing HVC of male (D) and female (E). F: Comparison of the number of cells double-labeled with BrdU and Hu per mm^2^ within the developing HVC between the two sexes. ** indicates *P*<0.001. Scale bar = 100 µm (A and B), and 50 µm (D, E and two inserts in A, B).

To determine the sexual difference in migration and cellular differentiation toward neurons, we compared the number of migrating BrdU-labeled cells along the radial glia fibers and the cells double-labeled with BrdU and Hu in the developing HVC between the two sexes, after the brain explants were cultured with BrdU within the first 24 h, and fixed 6 d after BrdU addition (the brain explants would become too thin to be cut for immunohistochemistry study, if cultured over 7 d). The results revealed that the number of BrdU-labeled cells along the radial glia fibers per mm^2^ in the developing male HVC (n = 21 from 5 birds) was significantly higher than that in the developing female HVC (n = 19 from 5 birds; male/female ratio = 1.68, t = 11.357, *P*<0.0001; Fig. 7A–C). There was a significantly larger number of double-labeled cells for BrdU and Hu in the male brain explants (n = 23 from 6 birds) than in the female explants (n = 21 from 5 birds; male/female ratio = 2.09, t = 16.435, *P*<0.0001; Fig. 7D–F).

### Influence of Estrogen and BDNF on the Cellular Proliferation and Differentiation in the Developing HVC and the Action between Estrogen and BDNF

To study the effect of estrogen and BDNF on the sexual differentiation of HVC, we first compared the distribution of BDNF and its specific receptor tyrosine kinase, TrkB in HVC at P15 and adult, and then added: 1) estradiol/estrogen antagonist (tamoxifen), 2) BDNF, or 3) both estradiol and BDNF antibody into the serum-free brain slice culture medium to analyze the changes of cell proliferation or differentiation.

### Distribution of BDNF and trkB in HVC

**Figure 8:**
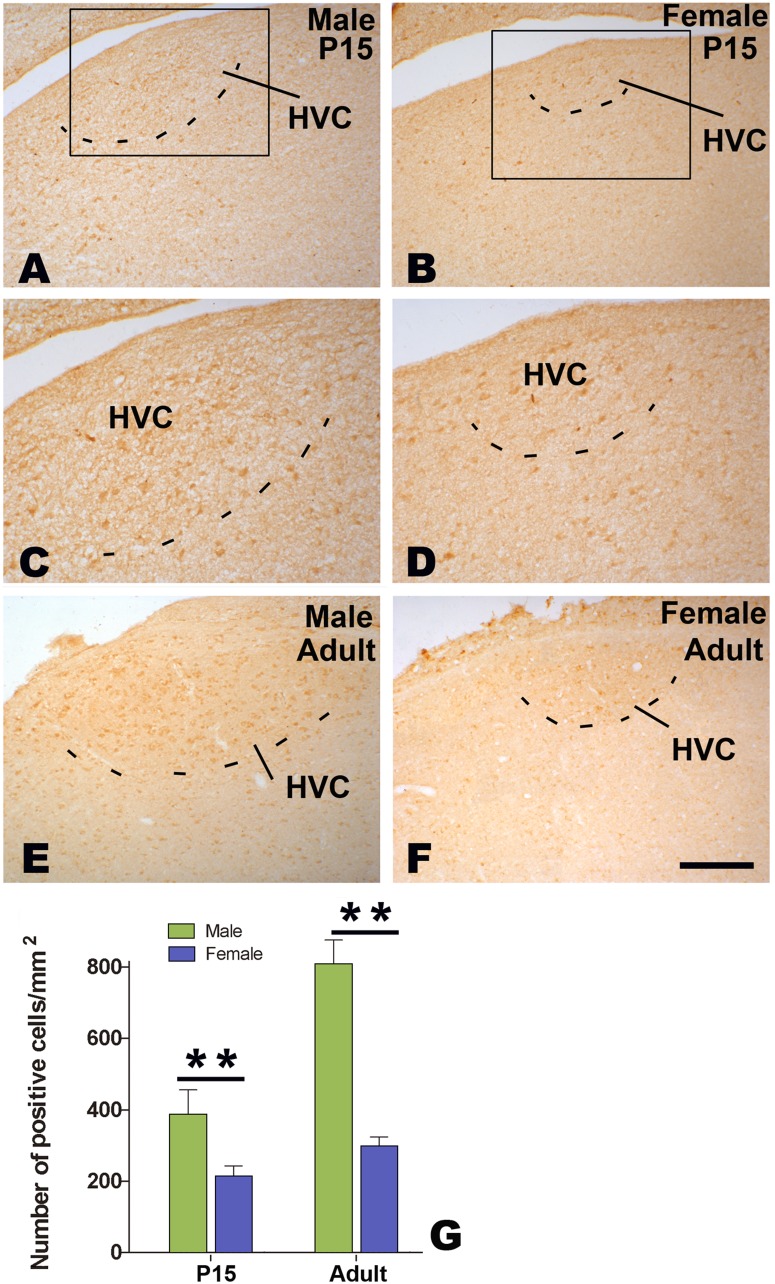
Distribution of BDNF-positive cells in HVC. BDNF-positive cells in HVC at 15 post-hatching days (P15, A–D) or at adulthood (E and F). Comparison of the number of BDNF-positive cells at P15 and adult (G). ** indicates *P*<0.001.

**Figure 9:**
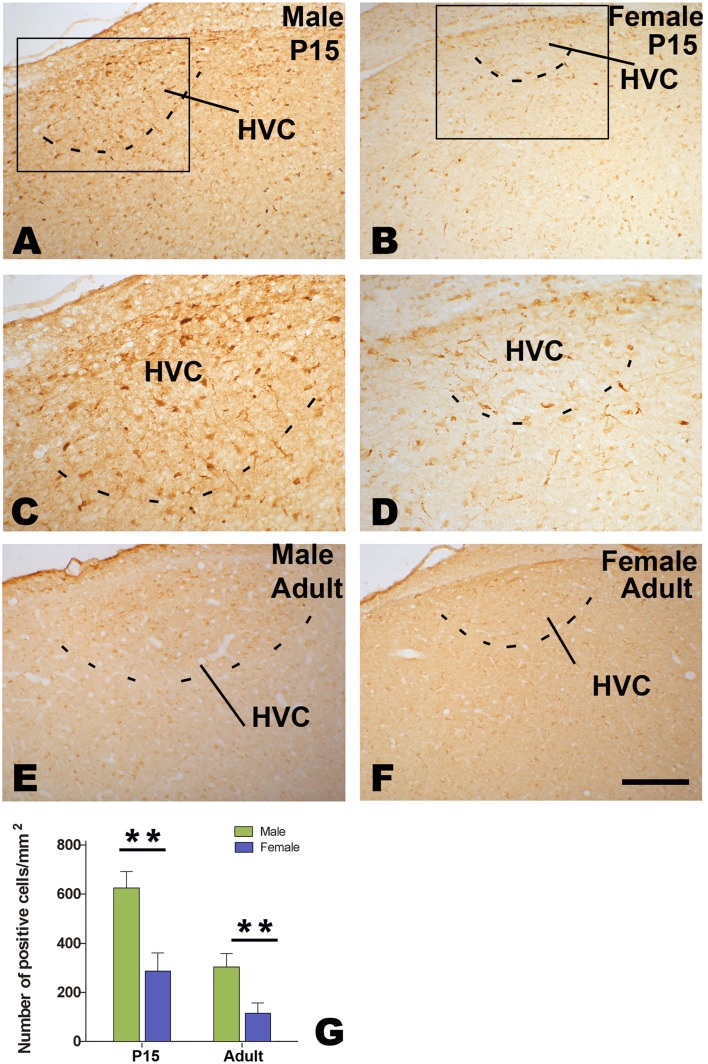
Distribution of TrkB-positive cells in HVC. TrkB-positive cells in HVC at 15 post-hatching days (P15, A–D) or at adulthood (E and F). Comparison of the number of TrkB-positive cells at P15 and adult (G). ** indicates *P*<0.001.

Many cells immunoreactive for BDNF or TrkB were seen in both male and female HVC (Figs. 8 A–F and 9 A–F). Compared to its surrounding areas, more BDNF immunoreactive cells were located in adult male or female HVC, and they thus could be distinguished out. Although female HVC was difficult to be identified on the basis of TrkB immunohistochemistry, it could be determined by reference to BDNF immunohistochemistry in each corresponding sections. The number of BDNF or TrkB cells per mm^2^ in the HVC showed significant differences between the two sexes (n = 5 for each group. BDNF: F_(1, 16)_ = 23.19, *P*<0.001; TrkB: F_(1, 16)_ = 23.19, *P*<0.001; Figs. 8G and 9G), and between the two ages (P15 and adult, statistical data not shown). There were no significant sex differences in BDNF or TrkB immunohistochemistry in the surrounding areas of HVC (Figs. 8A–F and 9A–F, data not shown).

### Effects of Estrogen or BDNF on the Proliferating Activity in the Ventricle Site of HVC and on Cell Differentiation within the Developing HVC

**Figure 10:**
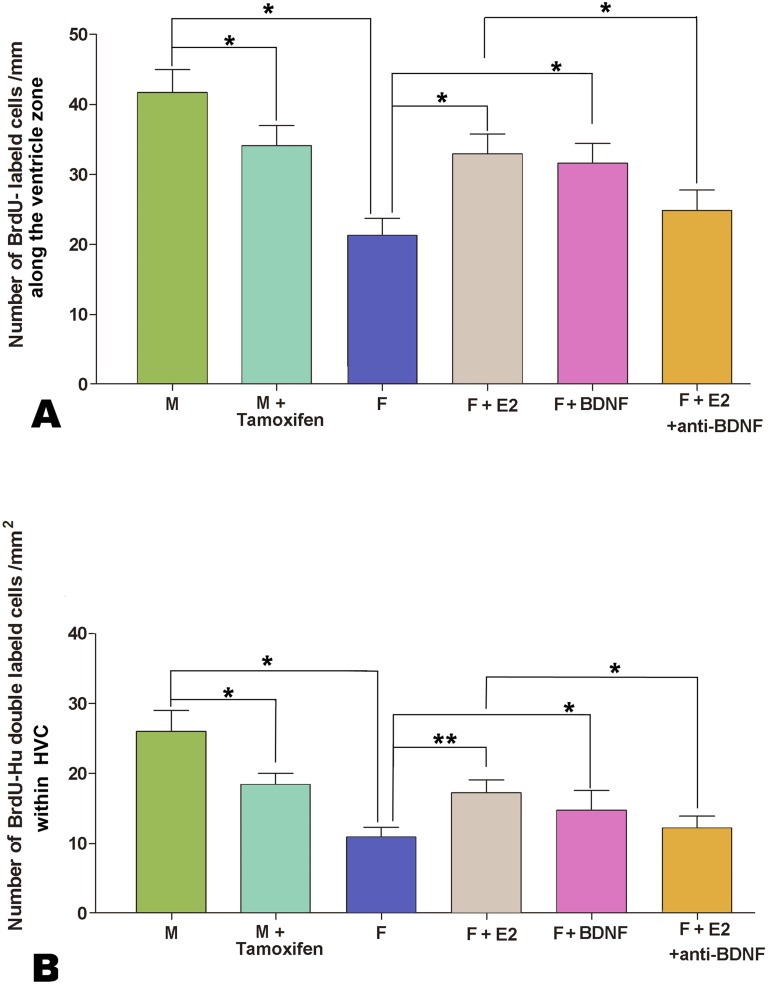
Influence of estrogen and BDNF on the cellular proliferation and differentiation in the developing HVC. A: Comparison of the number of BrdU-labeled cells along the ventricular zone per mm overlying the developing HVC. B: Comparison of the number of cells double-labeled with BrdU and Hu in the developing HVC. * indicates *P*<0.05, and ** indicates *P*<0.001.

One-way ANOVA analysis showed that the number of BrdU-labeled cells (per mm along the ventricular zone overlying the developing HVC) increased significantly, following 24 h culture of the female explants supplemented with estradiol (n = 36 from 7 birds) or BDNF (n = 33 from 6 birds), but decreased significantly after 24 h culture of the male explants supplemented with tamoxifen (n = 30 from 6 birds). However, the above increase did not occur if estradiol and BDNF antibody were added simultaneously (n = 35 from 7 birds) (F_(5, 205)_ = 14.409, *P*<0.001; Fig. 10A). We also examined the number of double-labeled cells for BrdU and Hu in the developing HVC, and found it increased significantly after 5 d culture of the female explants supplemented with estradiol (n = 36 from 7 birds) or BDNF (n = 33 from 7 birds). However, the increase of the above number did not appear if estradiol and BDNF antibody were added simultaneously (n = 35 from 7 birds) (F_(5, 198)_ = 11.963, *P*<0.001; Fig. 10B). In addition, the number of double-labeled cells for BrdU and Hu decreased significantly, following 5 d culture of the male explants supplemented with tamoxifen (n = 35 from 7 birds).

**Figure 11:**
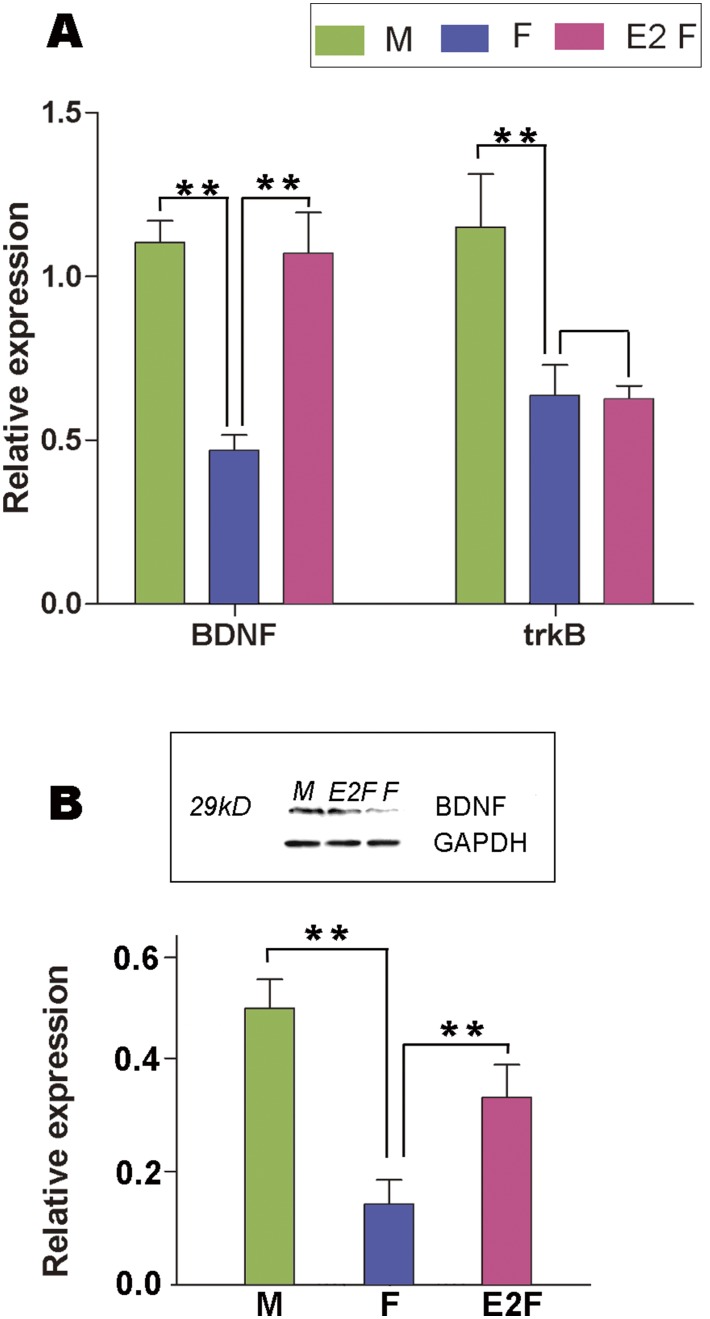
Quantitative-RT PCR or western-blot to show the expression of BDNF and its receptor trkB. Expression levels of BDNF and trkB mRNA (A) and BDNF protein (B) relative to GAPDH in the small brain slices containing male/female HVC at P15 or after the female brain slices containing HVC are cultured with the medium supplied with estradiol. ** indicates *P*<0.001.

To obtain more direct evidence concerning the interaction between estrogen and BDNF, we further examined the influence of E2 on the expression of BDNF and TrkB in the male and female explants containing the developing HVC. Our results indicated that BDNF mRNA increased significantly in the female explants after 48 hr culture with medium supplemented with exogenous estradiol (n = 6 for each group; BDNF: F_(2,17)_ = 15.699, p<0.001, Fig. 11A). Although TrkB mRNA level showed significant differences between the male and female explants, but did not change significantly after 48 hr culture of the female explants with medium supplemented with exogenous estradiol (F_(2,17)_ = 6.652, *P* = 0.966, Fig. 11A). In addition, the expression level of BDNF protein, which was assessed by using the relative optical density to GAPDH band (According to the manufacturer, this BDNF antibody recognized a specific band with 29 kD, which is a homodimer) relative to GAPDH also increased following the addition of estradiol into the culture medium for the female explants (F_(2,17)_ = 23.11, p<0.001, Fig. 11B).

### VEGF is not Involved in Estradiol-induced BDNF Up-regulation in the Developing HVC of Juvenile Bengalese Finches

**Figure 12:**
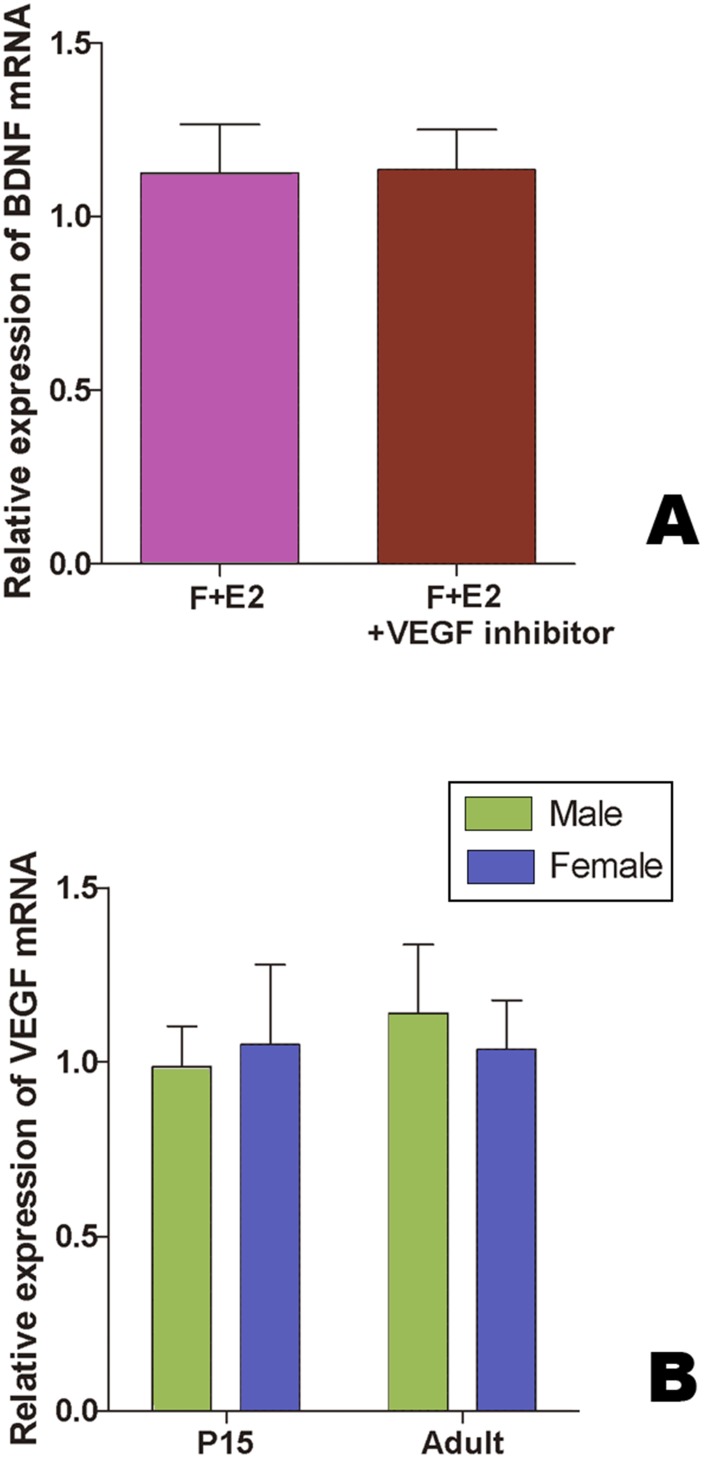
Quantitative-RT PCR to show BDNF and VEGF mRNA expression. A: The levels of BDNF mRNA relative to β-actin mRNA in HVC cultured in the medium supplied with estradiol only or with estradiol and VEGFR2 kinase inhibitor. B: The levels of VEGF mRNA relative to β-actin in male and female HVC at P15 and adult.

By using real-time RT-PCR, we found that, after 48 h culture, there were no significant differences in BDNF mRNA level relative to β-actin between the female explants with the serum-free culture medium supplied with estradiol only (n = 5 birds), and those supplied with estradiol and VEGFR2 kinase inhibitor (n = 7 birds, t = –0.048, *P* = 0.963, Fig. 12A). We then compared the levels of VEGF mRNA relative to β-actin in the brain slices containing HVC (they were cut as the method described above, Fig. 1A) between the two sexes at P15 and at adults (n = 5 birds for each group). The results showed no significant differences (F_(1, 16)_ = 0.168, *P* = 0.901, Fig. 12B).

**Figure 13:**
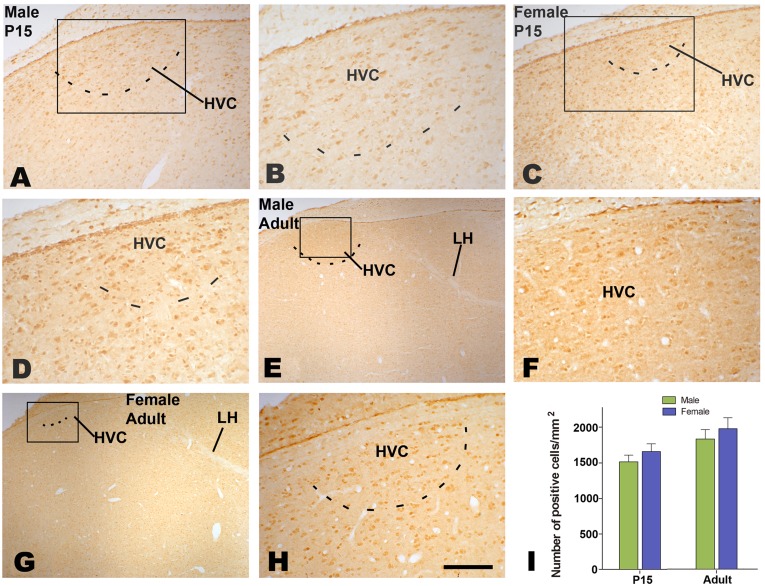
Distribution of VEGF-positive cells in the HVC. A–H: VEGF-positive cells at 15 post-hatching days (P15, A–D) or at adulthood (E–H). I: Comparison of the number of VEGF-positive cells in the developing HVC between the two sexes at P15 and adult. Scale bar = 200 µm (A and B), 500 µm (E, F), and 100 µm (C, D, G, H).

There were many VEGF-positive cells distributed in the developing and adult HVC (Fig. 13A–H). We compared the density of VEGF-positive cells in the developing and adult HVC between the males and females, and found no significant sexual differences (P15: n = 6 birds for each sex, F_(1, 20)_ = 3.983, *P* = 0.061, Fig. 13I).

**Figure 14:**
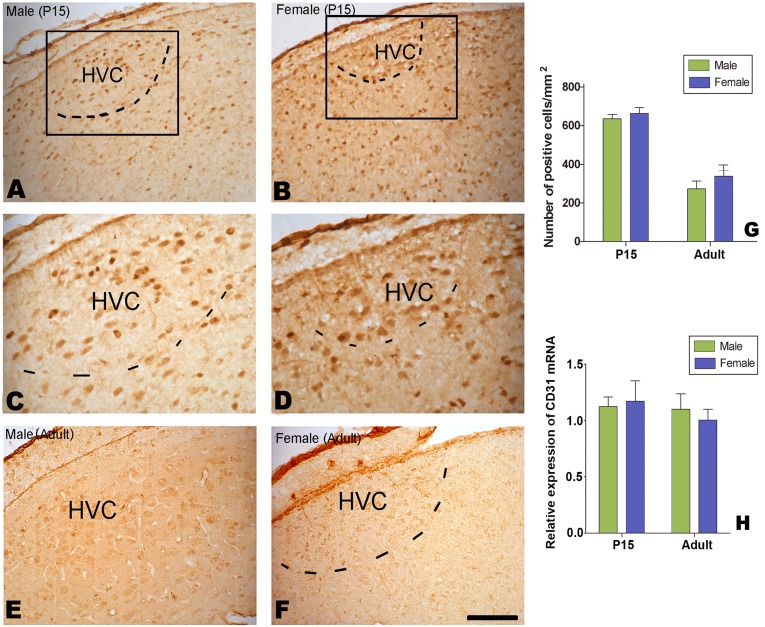
Distribution of Laminin-positive cells in HVC. laminin-positive cells in HVC at 15 post-hatching days (P15, A–D) or at adulthood (E and F). Comparison of the number of laminin-positive cells at P15 and adult (G). H: Expression levels of CD31 mRNA relative to β-actin in the male and female HVC.

Using the same antibody reported previously to identify endothelial cells positively immunohistochemically staining for laminin [22], we found many positive cells located in the developing HVC (Fig. 14A–D), but only weakly stained cells were observed in the adult HVC in both sexes (Fig. 14E and F). The density of laminin-positive cells (per mm^2^) in the HVC did not display any sexual differences at P15 or for adults (n = 6 birds for each group; F_(1, 20)_ = 0.264, *P* = 0.613; Fig. 14G).

As other antibodies specific for endothelial cells, such as CD31/PECAM-1, are not available at present time for Aves, we only compared CD31 mRNA in the HVC between the two sexes. We found no significant sexual differences in the expression level of CD31 mRNA relative to β-actin mRNA, which was extracted from the developing HVC (at P15) or adult HVC (n = 7 birds for each group; F_(1, 24)_ = 8.356, *P* = 0.325; Fig. 14 H).

## Discussion

### Effects on Sexual Differentiation of the Developing HVC

Our *in vivo* study showed that there were sex-specific cellular proliferation in the ventricle zone overlying the developing HVC and neuronal recruitment into HVC, and they were further confirmed by our *in vitro* experiments. We also showed that co-culturing of male and female brain tissues could result in a significant “masculinizing” of female proliferation, and that the sex differences in cellular proliferation and differentiation were estrogen sensitive, mediated by BDNF. Finally, our study indicated that the source of BDNF in HVC was not endothelial in difference to adult canary HVC.

Although some of the above results were first reported, our study was largely in agreement with several previous reports: 1) Cellular proliferation in the ventricular zone at the brain levels where the HVC is located is higher than at the other brain levels in juveniles [19], [35], [36]. 2) BDNF protein is distributed in HVC at P20 [37], and TrkB labeling is already distinct in HVC at P30 [31]. 3) The expression of BDNF mRNA increases significantly within 24 hr, following systemic estrogen treatments in male or female HVC at P15 and P20–25 [38], [39]. In addition, HVC location and size shown by backfilled Area X-projecting cells at P15, as shown in Fig. 1A–C, were largely similar to those shown by other methods, such as the distribution of androgen receptor mRNA around the same age in the zebra finch [38]. Thus, although the location and the size of juvenile HVC are difficult to be determined, especially in females, this problem could be resolved to some degree with the help of neural tract-tracing.

Owing to the limitation of practical operation, our small cut brain slices included not only HVC, but also its surrounding areas. As no significant sexual differences have been reported previously in the surrounding areas of HVC, as seen in the present study (such as the distribution of BDNF, TrkB, VEGF and laminin), the above sample should not affect our conclusion on the sexual differences of HVC. In addition, considering that HVC is substantially larger in males than in females (at least 3 times larger in volume) [1], [26], it is reasonable to conclude that the total number of examined immunoreactive cells in HVC is greater in males compared to females, if the density of the examined immunoreactive cells is higher in males than in females.

It is generally known that newborn cells originate in the proliferative ventricular zone adjacent to the lateral ventricles [40]–[42]; however, the exact location of HVC progenitors is not clear. Although lineage analysis using a library of retroviral vectors has demonstrated that HVC progenitor cells originate in the ventricular zone just overlying the developing HVC of juvenile birds [18], some cells have also been reported to be derived from the ventricle sites that are far away from the ventricular zone overlying their terminal locations [43]–[45]. For example, some neurons in the chick pallium have been observed to be generated in a paleo-striatal ventricular region, and migrated tangentially, rather than radially, to the pallium [46]–[47]. It is thus probable that HVC progenitors might be produced in other site(s). However, as a previous report demonstrated that newborn cells in the pallium are at least generated at the ventral hot spot [48], we only determined whether the dorsal hot spot is a potential resource of HVC progenitors.

In addition, the sex ratios of several measurements examined in the present study kept continual increase (including the number of proliferating cells in the ventricular zone overlying the developing HVC, the number of cells labeled with [^3^H]-thymidine along radial glia fibers, and the number of doubled labeled cells for [^3^H]-thymidine and Hu or NeuN). As previous reports have excluded post-mitotic divisions and cell death from being involved in the sexual differentiation of song control nuclei [49], the continually increasing ratios demonstrated that, in addition to cellular proliferation in the ventricular zone, cell migration and differentiation also contributed to the sex differences of HVC (if so, then there might not be continual increases).

It has been demonstrated that cells in or near song control nuclei autonomously synthesize estrogen at higher levels in cultured male brain slices of juvenile zebra finches than in cultured female slices, and this brain-derived estrogen is both necessary and sufficient for triggering formation *in vitro* of a male-specific synaptic connection [34]. Using the same culture system, which could prevent the endocrinological antagonism inherent in whole-animal manipulations, we replicated almost all of the main results obtained *in vivo*. Our data indicated that, similar to sex differences in the male-specific connection [34], sex differences in cell proliferation, migration and differentiation were also independent of gonad steroid hormones.

### Effects of Estrogen and BDNF on the Sexual Differentiation of HVC

Our co-culture of male and female slices containing the developing HVC in the same well indicated that the presence of male but not female tissue had significant effects on the masculinization of female HVC regarding cell proliferation, migration and differentiation, suggesting that the male tissue might be producing some diffusible substances necessary and sufficient for masculine development. Our following study confirmed that these diffusible substances at least, if not all (other factors might also be involved [50]), included estrogen and BDNF. Our study also revealed that after estradiol or BDNF was added to the culture medium for 24 h, the number of cells labeled with BrdU significantly increased, and such an increase could be blocked by BDNF antibody. In addition, the number of BrdU cells decreased significantly, following the addition of estrogen antagonist, tamoxifen, in the cultured male brain slices. These results were consistent with a previous report to indicate that male-specific synaptic connection can be stimulated in female slices if they are co-cultured with male slices or exposed to estrogen, but this can be inhibited by adding tamoxifen (the same catalog number as in the present study) into the cultured male brain slices [34].

Finally, we found that BDNF mRNA, but not TrkB mRNA, increased significantly in the female explants after 48 hr of culture with exogenous estradiol. Our data were in line with a previous report, which demonstrated that treatments with 17β-estradiol or testosterone increase BDNF levels, which finally promotes the addition of new neurons to adult female HVC [20], [38].

Estrogen has reported effects on gliogenesis, process outgrowth and neuronal migration, but not on neuronal proliferation, including reports on adult HVC [51]. In contrast, BDNF has been reported to have effects on cell proliferation [52], [53], the survival of some neuronal types [53]–[55] and cell differentiation [56], [57]. As a motif containing the canonical estrogen response element exists in the BDNF gene [58] and estrogen receptors colocalize to the same cells expressing BDNF and its receptor TrkB [59], [60]. Although these reports were from mammals, they might be also applied to songbirds. If so, these would be a biological basis for the interaction of estrogen and BDNF. Thus, even if gonad or brain-derived estrogens have no direct effect on cell proliferation in the ventricular zone, they could have a direct effect on BDNF.

However, it should be noted that the BDNF gene is not the sole target gene of estrogen, and the BDNF gene possesses several other promoters, including those binding to other steroid hormones and some signaling molecules such as cAMP [61], [62]. Therefore, other actions for either estrogen or BDNF exist that are independent of each other. This might be a reason why the estrogen-induced effects on cell proliferation and cell differentiation were not completely blocked by the addition of the BDNF antibody (Fig. 10A and B).

Our study also revealed that a Z-linked gene, a receptor for BDNF (TrkB) whose expression was independent of estrogen, had higher expression levels in the male developing HVC than in the female HVC. This is in accordance with a previous report indicating sexual differences in TrkB expression in the HVC at six days post-hatching when neither androgen nor estrogen receptors are expressed [64]. These data suggest that local estrogen-dependent effects cannot be responsible for the initial sexual dimorphism of TrkB expression in the HVC. Thus, in addition to steroid hormones, sex-linked genes might also initiate sex differences in brain development [63]. This assumption is of interest because it would help resolve the question of why the addition of estrogen could not fully masculinize song control nuclei (exclude genetic actions) [8], [64], [65], [66]. Using an Affymetrix GeneChip Chicken Genome Array (containing approximately 30,000 genes), we are now attempting to identify more sex-linked genes and other genes than BDNF- and estrogen-related genes that are involved in sex differences. We believe that these efforts will more clearly elucidate the mechanism of sexual differentiation of song nuclei.

### VEGF Pathway is not Involved in the Upregulation of Estradiol-induced BDNF in the Developing HVC

Testosterone-induced neuronal recruitment to the adult HVC in the female canary is ascribed to testosterone-induced increases of VEGF and its receptor expression, which, in turn, stimulates the proliferation of endothelial cells and thus greater BDNF production by endothelial cells [21], [22]. Our present results on the juvenile, developing HVC, however, differed from the above reports in several ways. 1) Our estrogen-induced up-regulation of BDNF mRNA could be detected just 48 hr after estrogen treatment but two weeks after testosterone treatment in adult HVC. 2) Testosterone-induced angiogenesis and neuronal recruitment could, but our BDNF up-regulation could not, be blocked by VEGF receptor inhibitor. These differences might be caused by the fact that, unlike in adult female HVC, our estrogen-induced BDNF up-regulation is not mediated by VEGF and its further induction of the proliferation of endothelial cells instead resulted from a direct estrogen action to BDNF. On the other hand, our results revealed that the amount of VEGF mRNA expressed in the developing HVC and its adjacent area did not display any significant sex differences, neither did the distribution of VEGF and laminin-expressing cells in the developing HVC, supporting the above conclusion that VEGF and endothelial cells are not involved in the observed estrogen-induced BDNF up-regulation.

**Figure 15:**
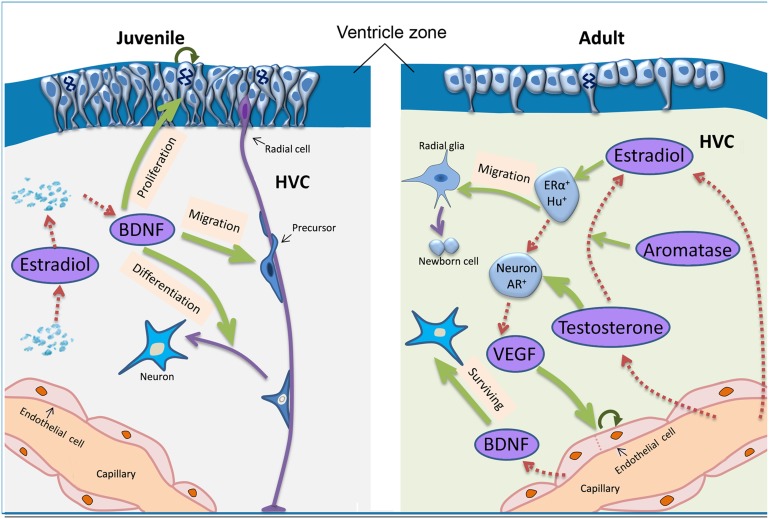
Summary of the assumed action routes of estradiol/testosterone on cell proliferation, migration and differentiation in adult (right) and juvenile (left) HVC. The action routes in adult HVC are discussed in a previous report (Louissaint et al., 2002). Note that there are substantial differences in the resources (shown by dashed brown arrows) of BDNF (from endothelial cells or not) and estradiol/testosterone (from capillary or from autonomous synthesis in local brain), and their actions during sexual differentiation of song control nucleus (shown by green arrows) between adult and juvenile birds. Curved arrows indicate some proliferating cells. Abbreviations: AR, androgen receptor; ER, estrogen receptor; Hu, Hu protein; VEGF, vascular endothelial growth factor; BDNF, brain-derived neurotrophic factor.

Given the above data, our study prompts us to suggest that the mechanism through which steroid hormones induced upregulation of BDNF and sex differences in cellular proliferation in the ventricular zone overlying the HVC might differ largely between adult and juvenile HVC (summarized in Fig. 15). Actually, unlike the present report on the juvenile birds at P15, cell proliferation in the ventricular zone overlying HVC or new-born cells recruitment into HVC in adult canaries showed no differences from those in the surrounding brain areas of HVC [67]. In addition, there is a greater number of proliferating cells in the ventricular zone underlying the developing HVC than in adulthood in the zebra finch, and there are sex differences in cell proliferation activity in the juvenile ventricular zone but not in the adult zone [35].
